# Real-Time Interface-Opening Detection for Nickel Starting Sheet Pre-Stripping Based on an Improved YOLO11n

**DOI:** 10.3390/s26144640

**Published:** 2026-07-22

**Authors:** Lei Wang, Junfeng Sun

**Affiliations:** 1 Faculty of Metallurgical and Energy Engineering, Kunming University of Science and Technology, Kunming 650093, China; wanglei6@stu.kust.edu.cn; 2 Nickel Smelter, Jinchuan Group Nickel and Cobalt Co., Ltd., Jinchang 737100, China; 3 Faculty of Mechanical and Electrical Engineering, Kunming University of Science and Technology, Kunming 650504, China

**Keywords:** nickel starting sheet, pre-stripping, interface opening, YOLO11n, industrial vision, real-time detection, PLC control

## Abstract

Real-time recognition of the transient interface opening between a nickel starting sheet and its titanium starter plate is required to stop high-frequency tamping at the correct instant. This study proposes Yolov11_tapcheck, a deployment-oriented detector based on YOLO11n, and implements a field real-time detection system for nickel starting sheet pre-stripping. To address small target scale, weak texture, reflective background interference, and motion blur, the model introduces efficient multi-scale attention (EMA) and reconstructs the neck as a BiFPN-inspired bidirectional feature-fusion path. A tapcheck dataset was established from production-line images, and the method was evaluated with image-level accuracy, latency, and field control metrics. Compared with the original YOLO11n, Yolov11_tapcheck improved mAP50 from 95.5% to 98.1% and F1 from 93.7% to 97.5%. The average model-pipeline latency was 7.1 ms; when MQTT delivery, OPCSERVER writing, PLC feedback timestamp comparison, and 1 ms polling were included, the average frame-to-PLC acknowledgement latency was 10.24 ms, with a maximum of 11.58 ms, remaining below the 13.333 ms frame interval in 1000 logged samples. Two field stages further showed that the opening success rate for cycles 3–8 increased from about 60–65% to more than 90%, while opening detection success increased from 90% to 100%. These results indicate that the proposed method can support real-time opening recognition and closed-loop control in nickel starting sheet stripping.

## 1. Introduction

Electrolytic processing is a major route for producing high-purity non-ferrous metals such as nickel, copper, and cobalt. In nickel electrowinning, the deposited nickel starting sheet must be detached from the titanium starter plate before subsequent stripping and grouping. Because the interfacial adhesion is affected by electrolysis conditions, surface condition, pickling, and mechanical contact, reliable opening formation is difficult to guarantee by fixed-time tamping alone.

**Figure 1:**
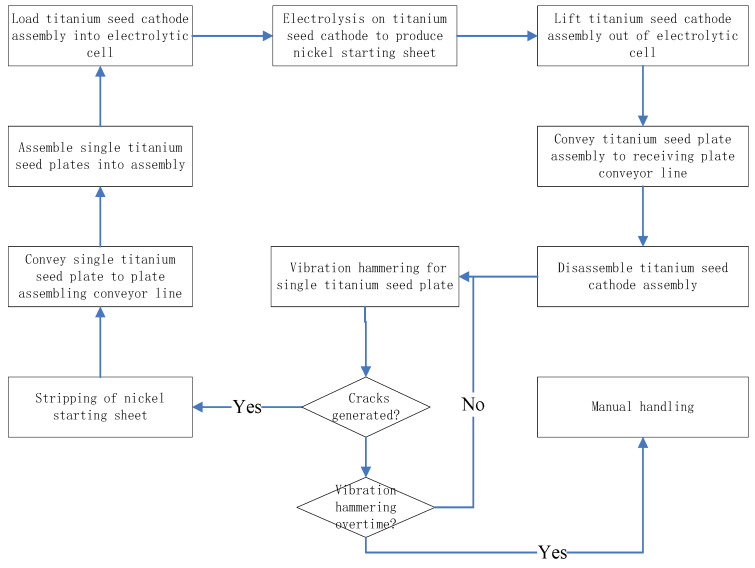
Nickel electrolysis production-line process flow, including tamping-based interface-opening formation, stripping, and regrouping.

In the production line considered in this work, the titanium starter plate is tamped to create a visible separation gap between the nickel starting sheet and the titanium starter plate before stripping. This separation is called an interface opening in this paper. It is not a material crack in the nickel sheet; rather, it appears as a narrow, crack-like gap in the captured images. If the opening is insufficient, repeated tamping or manual intervention is required, which reduces throughput and may increase mechanical risk. The tamping frequency is approximately 1000 strokes per minute, so the opening state must be identified under fast motion and strong vibration. Figure 1 shows the process flow.

Before deployment of the proposed visual detection system, a baseline audit of 300 starting sheets from the same production line showed that 53 sheets required manual intervention because the opening state could not be reliably determined or the pre-stripping effect was insufficient. This corresponds to a manual-intervention rate of 17.7%. For these affected sheets, repeated tamping and manual handling increased the average processing time by approximately 42 s per sheet. These observations provide the quantitative motivation for online recognition of the interface-opening state.

The main contributions of this study are as follows:

(1) A multi-layer control architecture was developed to perform image acquisition, interface-opening detection, and production-line control in parallel.
(2) A Yolov11_tapcheck detector was designed by introducing EMA modules and a BiFPN-inspired bidirectional neck into YOLO11n for small, blurred, low-contrast interface openings.
(3) A field dataset was built from production-line images, and the model was evaluated using accuracy, false-positive and false-negative analysis, model-pipeline latency, and frame-to-PLC acknowledgement latency.
(4) Two field test stages were used to verify opening success, detection reliability, and visible starter-plate damage under production conditions.

## 2. Related Work

YOLO-based detectors and their variants have been widely applied to industrial, agricultural, transportation, and structural defect detection. Existing crack and defect studies commonly improve detection through lightweight backbones, attention mechanisms, feature-fusion redesign, and detection-head optimization. Representative examples include YOLO-KD and MGD-YOLO for road defects, YOLO11n-seg and YOLO-ERCD for surface and road cracks, OSCD-YOLO for open-pit mine-slope cracks, CLGDS and YOLO-based dynamic pyramids for bridge defects, RDLK-YOLO for pipeline defects, SCS-YOLO for crop stem cracks, PV-YOLO-type models for photovoltaic defects, SASED-YOLO for wind turbine blades, and LBA-YOLO or CGSW-YOLO for building and concrete cracks [1,2,3,4,5,6,7,8,9,10,11,12,13,14].

Most of these studies focus on static or quasi-static images where crack boundaries are relatively clear and imaging conditions are controllable. The present task differs in four aspects: the target is a transient interface opening rather than a structural crack; images are captured during high-frequency tamping; the target occupies a very small portion of the image; and the detector must be connected to MQTT, OPCSERVER, and PLC logic for real-time stopping.

Beyond surface-defect detection, real-time deep learning has also been used for process-state recognition in dynamic manufacturing. For example, temporal thermal data have been used to characterize fused filament fabrication states for in-process quality monitoring [15]. Such studies support the broader relevance of real-time visual or temporal monitoring for manufacturing control, while the present work focuses on visible-light opening detection and closed-loop tamping control. Table 1 summarizes the methodological gap.

## 3. System Architecture

**Figure 2:**
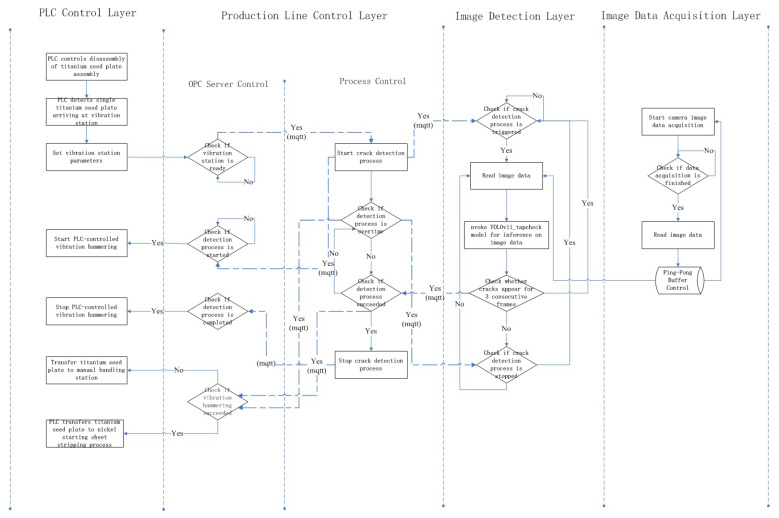
System control architecture linking PLC control, OPCSERVER communication, production-line control, image detection, and image acquisition. Solid arrows represent process and control-flow relationships, whereas blue dashed arrows represent MQTT message and data transmission. Arrowheads indicate the direction of process execution or data transmission, and vertical dotted lines separate the functional layers.

The system uses a layered architecture to complete interface-opening detection and production-line control in real time. The image acquisition station and the tamping station are the same physical station. Two industrial cameras and two light sources acquire images from the front and back sides of the titanium starter plate. Figure 2 shows the system control architecture, including PLC control, OPCSERVER communication, production-line control, image detection, and image acquisition.

**Figure 3:**
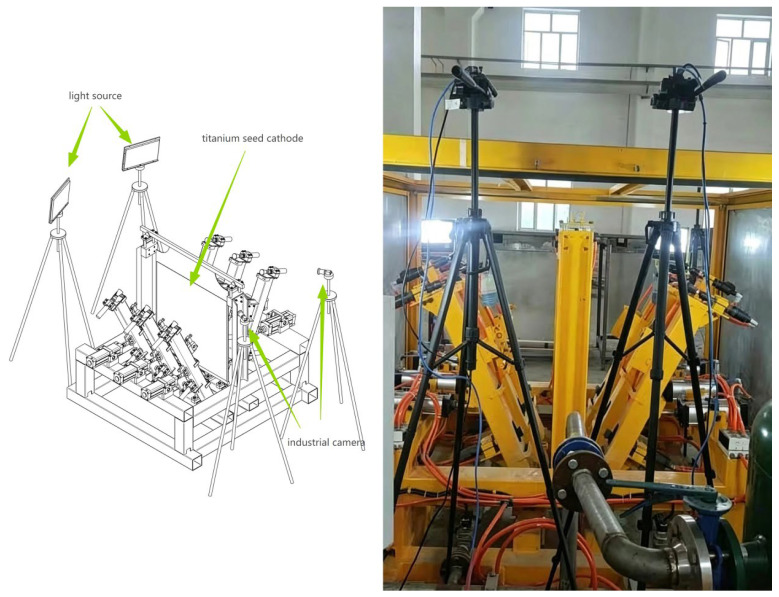
Field image acquisition station with two cameras and supplementary illumination at the tamping station.

The image acquisition layer controls Daheng MER2-160-75GC industrial cameras (China Daheng Group, Inc. Beijing Image Vision Technology Branch, Beijing, China) through Gigabit Ethernet. The raw camera setting is 75 FPS, 1440 × 1080 pixels, global shutter, 20 us exposure, and 8 dB gain. The detailed optical and image-acquisition parameters are listed in Table 2. To reduce the exposure time while maintaining sufficient brightness, two 100 W Bull LED floodlights are used for supplementary illumination, as shown in Figure 3. The camera-lens combination is a MER2-160-75GC camera with an HN-P-0828-6M-C2/3 lens, focal length 8 mm, and an approximate working distance of 1 m. The cameras operate in continuous acquisition mode, while the effective detection window is controlled by production-line state signals. The acquired stream is written into a ping-pong buffer for the detection layer.

The image detection layer starts after receiving a start-detection message from the production-line control layer. It reads the latest valid frame from the ping-pong buffer and performs inference with Yolov11_tapcheck through Microsoft.ML.OnnxRuntime.Gpu. Once an interface opening is detected, the result and timestamp are sent to the production-line control layer through MQTT.

The production-line control layer reads line state with an approximately 1 ms period on the industrial computer using a C# timing thread based on CPU ticks. This polling period is therefore implemented by the industrial computer software rather than by a PLC cyclic interrupt task. When a titanium starter plate reaches the tamping station and tamping begins, the control layer notifies the detection layer to start detection. After receiving a valid detection result, it updates the PLC data area through OPCSERVER and commands tamping to stop.

## 4. Materials and Methods

### 4.1. Problem Description and Imaging Conditions

**Figure 4:**
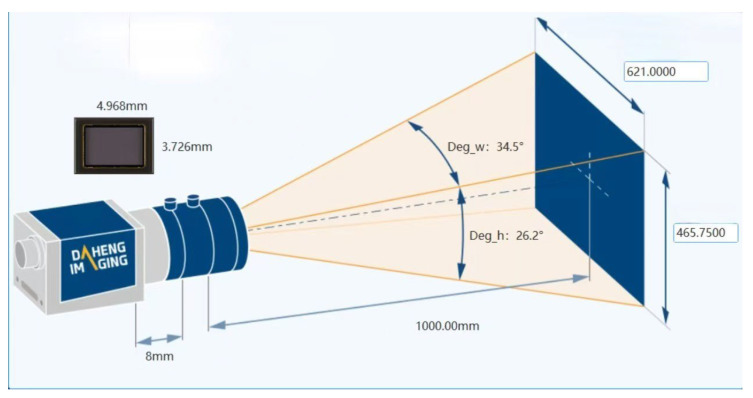
Field-of-view calculation for the image acquisition equipment.

**Figure 5:**
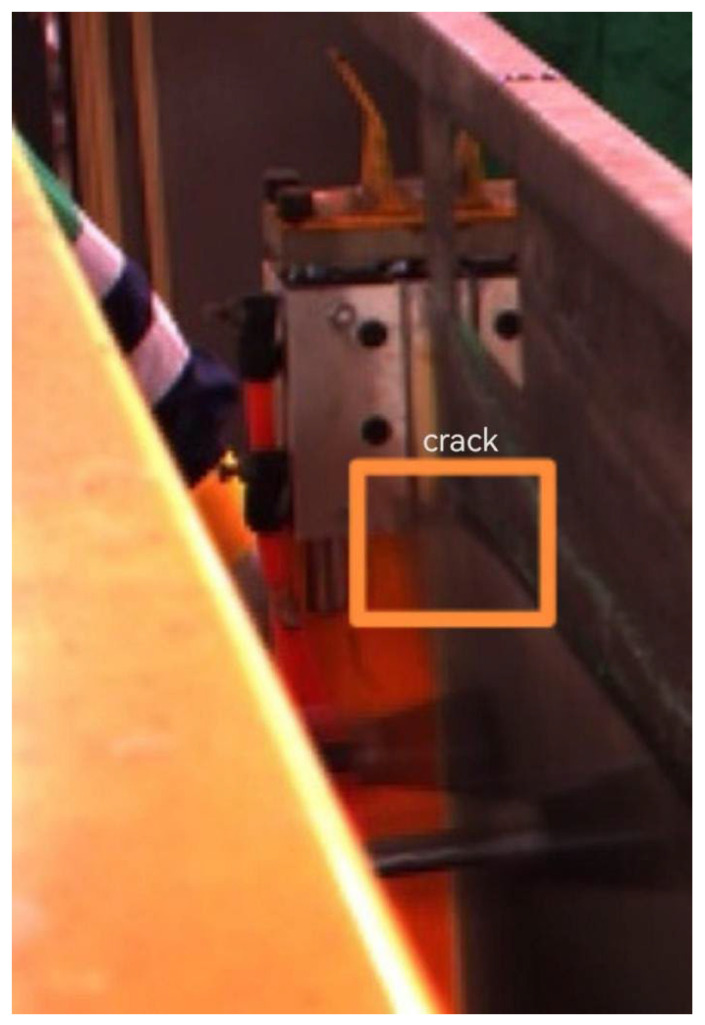
Low-contrast interface-opening edge under field illumination.

After the image detection layer obtains image data, the model determines whether the image contains an interface-opening target. The field-of-view calculation for the acquisition equipment is shown in Figure 4. Because image acquisition occurs during tamping, the opening region is affected by motion blur. The edge-to-background contrast is particularly low, as shown in Figure 5. The target occupies only a small area in the full image and appears under diverse field conditions. These conditions require a detector that can preserve weak local features while remaining fast enough for online control.

### 4.2. Baseline YOLO11n

YOLO11n is the nano detector in the Ultralytics YOLO11 software release (version 8.3.28) [16]. It follows a backbone–neck–head structure and is suitable for edge-side or real-time tasks because of its compact parameter count and low computational cost. The backbone extracts multi-scale features using Conv, C3k2, C2PSA, and SPPF modules. The original neck uses a lightweight PAN-style path to aggregate features from different scales, and the detection head predicts object location and class probabilities in an anchor-free manner.

Although YOLO11n is fast, the original PAN neck does not sufficiently emphasize weak, blurred, and small opening features in the present industrial scene. Therefore, this work keeps the lightweight backbone and reconstructs the feature-fusion part for the opening-detection task.

### 4.3. Yolov11_Tapcheck

#### 4.3.1. EMA Efficient Multi-Scale Attention

EMA is a lightweight CNN attention module that preserves channel dimensionality while using channel grouping, axis-wise global pooling, local 3 × 3 convolution, and cross-spatial interaction [17]. In this task, EMA is used to enhance small edge and texture evidence and suppress reflective background interference.

#### 4.3.2. BiFPN-Inspired Bidirectional Multi-Input Feature-Fusion Neck

For a 640 × 640 input, the reconstructed neck operates on three detection scales: P3, P4, and P5, corresponding to feature-map sizes of 80 × 80, 40 × 40, and 20 × 20 and strides of 8, 16, and 32, respectively. The top-down path propagates deep semantic information from P5 to the higher-spatial-resolution P4 and P3 branches. Specifically, the deep P5 feature is upsampled by a factor of two and concatenated with the lateral P4 feature, producing a 40 × 40 × 384 tensor. A 1 × 1 convolution compresses this tensor to 128 channels, followed by C3k2 refinement. The resulting top-down P4 feature is subsequently upsampled and concatenated with the backbone P3 feature. The resulting 80 × 80 × 256 tensor is compressed to 64 channels and refined by C3k2 to generate the P3 branch.

The bottom-up path propagates fine-grained edge and localization information from P3 toward the lower-spatial-resolution P4 and P5 branches. After stride-2 downsampling, the P3 output is jointly concatenated with the lateral P4 feature and the T5 feature, forming a three-input 40 × 40 × 384 fusion node. Similarly, the downsampled P4 output is concatenated with both the backbone P5 feature and the deep P5 semantic feature, forming a three-input 20 × 20 × 768 fusion node. Each bottom-up fusion node is followed by 1 × 1 channel compression and C3k2 refinement.

The proposed design is inspired by the bidirectional cross-scale topology of BiFPN but does not employ learnable normalized fusion weights. Instead, concatenation-based multi-input fusion is used to retain complementary information from different paths, while 1 × 1 convolutions control channel growth and computational cost.

#### 4.3.3. Network Reconstruction

**Figure 6:**
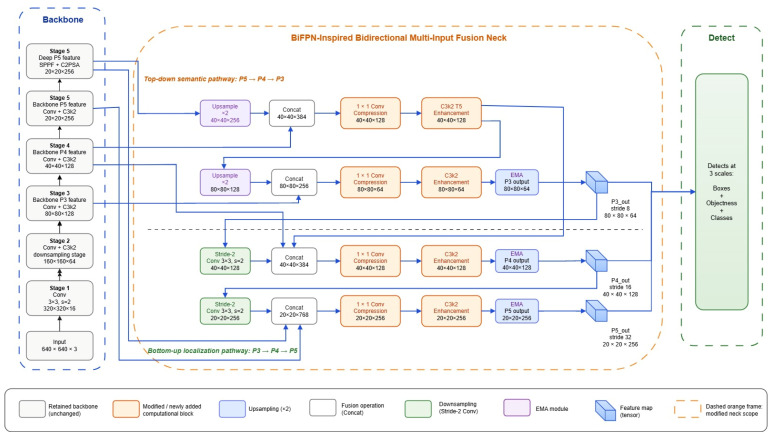
Network architecture of the proposed Yolov11_tapcheck model.

Yolov11_tapcheck retains the YOLO11n backbone and reconstructs the neck using bidirectional cross-scale transmission, multi-input concatenation, lightweight channel compression, and output-level attention enhancement, as shown in Figure 6. For a 640 × 640 input, the backbone provides the P3 feature at 80 × 80 × 128, the P4 feature at 40 × 40 × 128, the P5 feature at 20 × 20 × 256, and a deeper P5 semantic feature at 20 × 20 × 256 after the SPPF and C2PSA modules.

In the top-down path, the deep P5 feature is first upsampled and fused with P4 to generate an intermediate top-down P4 representation. This representation is then upsampled and fused with P3 to produce P3_out. In the bottom-up path, P3_out is downsampled and jointly fused with the backbone P4 feature and the T5 feature to produce P4_out. Subsequently, P4_out is downsampled and jointly fused with the backbone P5 feature and the deep P5 feature to produce P5_out.

The final output tensors are P3_out at 80 × 80 × 64 with stride 8, P4_out at 40 × 40 × 128 with stride 16, and P5_out at 20 × 20 × 256 with stride 32. EMA modules are applied only after the completed P3, P4, and P5 fusion branches rather than to redundant intermediate features. This placement enhances detection-ready edge and texture representations while avoiding unnecessary attention computation.

### 4.4. Dataset

The tapcheck dataset was collected from the production line at different times of day to include variation in workshop illumination and background interference. Raw camera frames were acquired at the field station and stored or resized for model training and testing. The dataset contains 3155 images and was split at the starting-sheet-event level into training, validation, and test subsets at an approximate ratio of 70:15:15. The split contains 2208 training images, 473 validation images, and 474 test images. Each subset includes both opening-containing and opening-free images. The detailed dataset composition and leakage-control protocol are summarized in Table 3.

Bounding boxes were created using LabelImg 1.8.6. A box encloses the visible separation gap and its immediately adjacent edge. Reflections, stains, and edge textures without a continuous physical separation are treated as negative samples. Ambiguous frames were reviewed manually and excluded when the opening boundary could not be determined reliably. Two trained annotators labelled the data independently, and disagreements were adjudicated by an experienced engineer. The pre-adjudication agreement was 94.6% at IoU ≥ 0.80. For descriptive analysis, micro-, medium-, and large openings are defined as visible gap widths of <12 pixels, 12–24 pixels, and >24 pixels, respectively. These size groups were used only for descriptive analysis and representative visualization. All annotated targets shared a single class label, interface opening, during model training and evaluation.

To reduce leakage, images were first grouped by starting-sheet event and continuous acquisition sequence before splitting. A perceptual-hash screen was also used to identify near-duplicate frames. No frames from the same sheet event or continuous sequence were distributed across training, validation, and test subsets.

**Figure 7:**
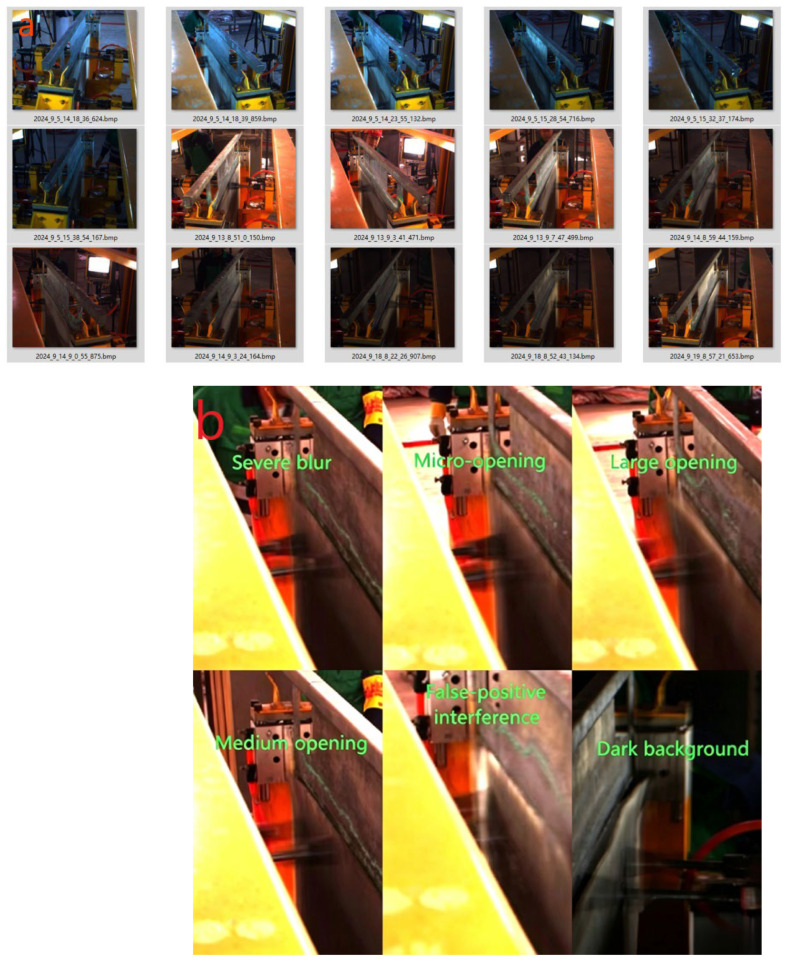
Representative samples from the constructed tapcheck dataset: (**a**) original field images collected under different illumination and background conditions; (**b**) representative challenging cases, including severe motion blur, micro-openings, medium openings, large openings, stain-like interference, and dark background samples. The size groups are used only for visualization and descriptive analysis; all annotated targets share a single class label, interface opening.

Representative samples from the constructed tapcheck dataset are shown in Figure 7. Figure 7a presents original field images collected under different illumination and background conditions, while Figure 7b shows representative challenging cases, including severe motion blur, micro-openings, medium openings, large openings, stain-like interference, and opening-free samples.

### 4.5. Training Setup and Evaluation Metrics

Training and evaluation were performed on Windows 10 with 32 GB RAM, an NVIDIA RTX 4070 SUPER GPU, and an Intel Core i9-14900K CPU. The software environment included Python 3.11.5, PyTorch 2.5.1, CUDA 11.8, and Ultralytics 8.3.28. The reproducible training and inference settings are summarized in Table 4. All models were trained for 200 epochs using SGD optimization, with momentum of 0.937, weight decay of 0.0005, an input image size of 640 × 640, 3 warm-up epochs, and an automatically selected batch size of 32. Images were resized by letterboxing to preserve aspect ratio. Data augmentation included HSV adjustment, translation, scale augmentation, horizontal flipping, random erasing, and mosaic augmentation. Accuracy comparisons were repeated with five random seeds (0, 42, 123, 2025, and 3407), and the same training protocol was used for the baseline, comparison models, and ablation variants.

Precision (P), recall (R), mAP50, mAP50-95, and F1 were used to evaluate detection accuracy. F1 is the harmonic mean of precision and recall: F1 = 2PR/(P + R). Because this system detects a single target type, namely the interface opening between the titanium starter plate and the nickel starting sheet, AP and mAP are equivalent for the single class. The term mAP is retained following common object-detection reporting convention, and F1 and mAP are used as the main performance indicators.

## 5. Results

### 5.1. Comparison with the Baseline Model

To evaluate the effectiveness of the model improvement, Yolov11_tapcheck was compared with the original YOLO11n and additional lightweight detectors on the event-separated test set. Table 5 reports mean accuracy over five training runs, together with model complexity and latency. Yolov11_tapcheck improved mAP50 from 95.5% to 98.1% and F1 from 93.7% to 97.5% compared with YOLO11n, indicating improved representation of blurred, weak-texture, and difficult opening samples.

Table 5 shows that Yolov11_tapcheck achieved the best overall accuracy among all compared detectors. Relative to the lightweight YOLOv8n, YOLOv10n, and YOLO11n baselines, the proposed model increased mAP50 by 2.7, 2.2, and 2.6 percentage points, respectively, and increased F1 by 3.7, 3.2, and 3.8 percentage points. The gain over YOLO11n is especially important because it uses the same base family and training protocol; therefore, the improvement mainly comes from the task-oriented attention and feature-fusion redesign rather than from changing to a larger backbone or a different detector family.

From an industrial-control perspective, the improvement in recall is more meaningful than a small isolated increase in precision. Compared with YOLO11n, recall increased from 0.941 to 0.971, which means that fewer formed openings are missed during the short detection window after tamping. At the same time, precision increased from 0.934 to 0.981, indicating that the additional feature enhancement did not simply make the detector more sensitive; it also reduced background-induced responses from reflections, stains, and weak edge-like textures.

The comparison with YOLO11s and RT-DETR-R18 further indicates that the proposed model has a better accuracy-efficiency balance for deployment. Yolov11_tapcheck achieved higher mAP50, mAP50-95, and F1 than YOLO11s while using 67.0% fewer parameters, 61.9% fewer GFLOPs, 65.8% less model storage, and 27.6% lower latency. Compared with RT-DETR-R18, it reduced parameters and GFLOPs by 84.5% and 85.6%, respectively, and reduced latency from 14.9 ms to 7.1 ms. These results suggest that the model improvement is not a brute-force gain from increased capacity, but a targeted enhancement for small, blurred, and weak-texture interface openings.

The additional computational cost over YOLO11n is limited. Parameters increased from 2.6 M to 3.1 M, GFLOPs from 6.5 to 8.2, GPU memory from 0.71 GB to 0.78 GB, and latency from 6.7 ms to 7.1 ms. This 0.4 ms latency increase is small relative to the 13.333 ms frame interval of the 75 FPS camera, while the accuracy and F1 improvements directly reduce the risk of missed or premature stop decisions. Therefore, Yolov11_tapcheck provides a more suitable trade-off for online pre-stripping detection than both the nano baselines and the larger comparison models.

### 5.2. Ablation Experiment

Ablation experiments were performed to evaluate both the contribution of the BiFPN-inspired bidirectional feature-fusion neck (BFFN) and the insertion positions of EMA. EMA was tested after the P3 branch, after the P3–P4 branches, and after the P3–P5 branches. BFFN alone improved mAP50 from 95.5% to 96.5%, mAP50-95 from 69.8% to 71.2%, and F1 from 93.7% to 94.5%. EMA improved the suppression of irrelevant texture, and the combination of P3–P5 EMA with BFFN produced the best overall result.

The EMA placement results in Table 6 show a clear multi-scale trend. Adding EMA only at P3 improved mAP50 from 0.955 to 0.964 and F1 from 0.937 to 0.954, indicating that attention on high-resolution shallow features is effective for fine opening edges. Extending EMA to P3–P4 and P3–P5 further increased mAP50-95 from 0.708 to 0.714 and 0.718, respectively, and raised F1 to 0.958 and 0.961. This gradual improvement suggests that the opening state is not represented by a single scale: shallow features preserve narrow edge details, whereas deeper features help suppress reflective background regions and ambiguous sheet textures.

BFFN alone produced a different type of improvement. It increased mAP50 from 0.955 to 0.965 and mAP50-95 from 0.698 to 0.712, showing that bidirectional feature flow helps localization and cross-scale feature reuse. However, its F1 score reached only 0.945, lower than the EMA-only P3-P5 configuration. This indicates that feature fusion improves geometric representation, but attention is still needed to emphasize the true interface-opening cues under motion blur and uneven illumination.

The best configuration combined BFFN with EMA at P3-P5. Compared with YOLO11n, this configuration improved precision by 4.3 percentage points, recall by 3.2 percentage points, mAP50 by 2.8 percentage points, mAP50-95 by 3.2 percentage points, and F1 by 3.8 percentage points. The combined result is also higher than either BFFN alone or EMA alone, which supports the complementarity of the two modifications: EMA recalibrates discriminative responses at multiple scales, while BFFN strengthens bidirectional information exchange between shallow edge features and deeper semantic features.

The ablation also shows that the performance gain is obtained with modest overhead. The final configuration added 0.5 M parameters, 1.7 GFLOPs, and 0.4 ms latency compared with YOLO11n. Because the largest accuracy gains occur in recall and F1, this overhead is acceptable for the pre-stripping task, where both missed detections and false triggers can affect mechanical action. The P3-P5 EMA plus BFFN setting was therefore selected as the final model because it gives the strongest and most balanced improvement without violating the real-time requirement.

### 5.3. False-Positive and False-Negative Analysis

**Figure 8:**
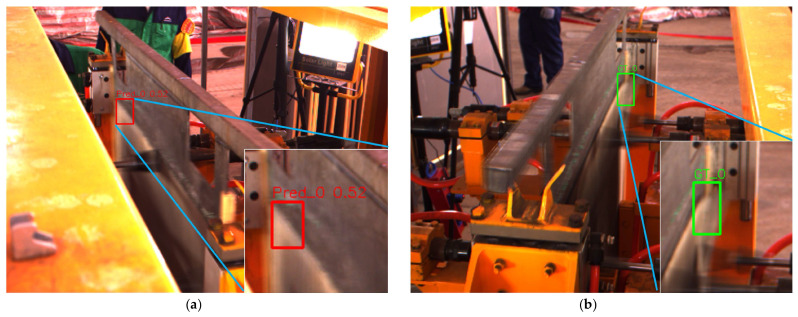
Representative detection examples and error cases: (**a**) false positive caused by stain-like edge texture; (**b**) false-negative.

A total of 474 event-separated test images were used for false-positive and false-negative analysis, including 292 opening-containing images and 182 opening-free images. The detailed false-positive and false-negative statistics are summarized in Table 7. As shown in Figure 8, a false positive occurs when the model predicts an opening although no effective opening is present, which may cause premature stopping. In contrast, a false negative occurs when an already formed opening is not detected, potentially resulting in continued tamping and increased mechanical risk.

### 5.4. Real-Time Verification

Real-time inference was evaluated on a platform equipped with an NVIDIA RTX 4070 SUPER GPU, an Intel Core i9-14900K CPU, and 32 GB of RAM. Using Ultralytics 8.3.28, Python 3.12.7, and Torch 2.5.1 + cu118, the average preprocessing time was 1.3 ms, inference time was 4.3 ms, and post-processing time was 1.5 ms. The resulting model-pipeline time was 7.1 ms, corresponding to approximately 140.8 frames per second, which is higher than the 75 FPS image-acquisition rate.

The complete system was implemented using C#, Microsoft.ML.OnnxRuntime.Gpu, MQTT, and OPCSERVER. At 75 FPS, the camera produces a new frame every approximately 13.333 ms. This frame interval is the available software-processing deadline; it is not the measured latency of an individual frame. A timestamp is generated only when a frame enters the detection stage. The detection result together with this timestamp is then sent to the production-line control layer through MQTT. The control layer writes the result to the PLC data area through OPCSERVER and compares the returned PLC timestamp with the original timestamp to evaluate the complete software-control processing time.

Table 8 reports the latency statistics of the model pipeline and the frame-to-PLC acknowledgement path. The model-pipeline time includes preprocessing, GPU inference, and post-processing. The frame-to-PLC acknowledgement time includes the model pipeline, result transmission, MQTT delivery, OPCSERVER writing, PLC feedback timestamp comparison, and 1 ms cyclic PLC-state polling. Camera exposure, image sensor readout, image transfer before the detection stage, frame-phase waiting, and mechanical actuator response were not included in this timing boundary.

For system-level timing, the acquisition layer supplies one frame every approximately 13.333 ms. When the detection layer starts processing a frame, it generates timestamp t0. After inference, the result and t0 are sent through MQTT to the production-line control layer, which updates the PLC data area through OPCSERVER. The returned PLC timestamp marks the end of the measured interval. Thus, the frame-to-PLC acknowledgement latency includes inference, result transmission, PLC writing, PLC feedback, and the 1 ms cyclic PLC-state read. It does not include camera exposure, sensor readout, image transfer before t0, waiting for the next frame, or mechanical actuator response.

The 1 ms PLC-state read was implemented by a C# thread combined with CPU tick timing on the industrial computer. Under the current hardware conditions, the timing resolution was approximately 20 us. Timing stability was evaluated from the logged timestamps before and after each PLC-state read. If the elapsed interval exceeded 1 ms, an overtime event was written to the log. No overtime event was observed during the 1000-sample full-speed verification.

**Figure 9:**
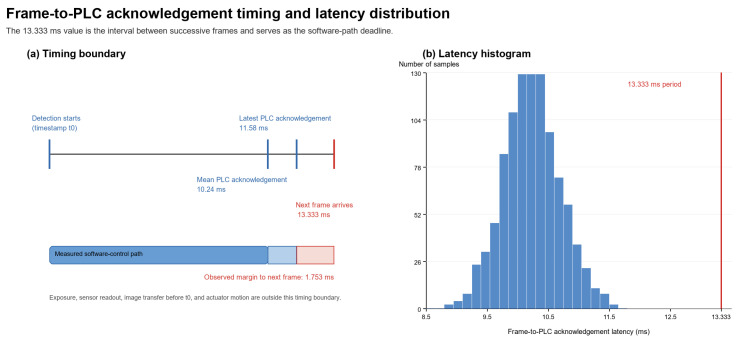
System-level timing definition and frame-to-PLC acknowledgement latency based on 1000 logged samples. (**a**) Measurement boundary from the start of detection to PLC acknowledgement; the 13.333 ms camera-frame interval is the software-path deadline. (**b**) Histogram of the measured frame-to-PLC acknowledgement latency. Camera exposure, pre-detection image transfer, and actuator response are excluded.

For the frame-to-PLC acknowledgement path, the mean latency was 10.24 ms, the standard deviation was 0.46 ms, the median was 10.21 ms, the p95 latency was 11.03 ms, the p99 latency was 11.34 ms, and the maximum latency was 11.58 ms. The maximum measured latency was therefore 1.753 ms shorter than the 13.333 ms interval before the next frame became available. Figure 9 defines this timing boundary and shows the latency histogram. The absence of samples at 13.333 ms is expected because this value is the frame-period deadline rather than a measured latency target.

## 6. Industrial Field Test and Application Effect

### 6.1. Test Purpose and Equipment

Field tests were conducted to verify both the feasibility of tamping-based opening pre-stripping and the real-time recognition capability of the proposed system. The test system included a tamping pre-stripping machine, a visual intelligent detection system, and an electrical control system. The visual system consisted of industrial digital cameras, illumination devices, an industrial computer, and the opening-recognition software.

### 6.2. Test Plan

The field test was divided into two stages. Stage I was conducted from 27 August to 26 September 2025, and Stage II from 27 October to 17 November 2025. In both stages, the opening recognition window was set to 1–8 s. If an opening was detected within this window, tamping was stopped; otherwise, the starting sheet was considered not to have reached effective pre-stripping.

Stage I included 612 starting-sheet tests. Based on problems exposed in Stage I, Stage II optimized the tamper type and layout, updated the recognition program, and extended the titanium starter-plate pickling time to 2 min 30 s. Stage II included 304 starting-sheet tests.

### 6.3. Test Results

The exact test counts and opening outcomes for the two field stages are summarized in Table 9. In Stage I, the first-cycle opening success rate was 3/102 (2.9%), the second-cycle success rate was 9/104 (8.7%), and the cycles 3–8 success rate was 253/406 (62.3%). The detection system identified 238 of 265 manually confirmed opening events, corresponding to 89.8%. These results show that tamping-based opening is feasible under certain conditions, but equipment parameters, recognition software, and process conditions still require optimization.

In Stage II, the tamper was changed from AT-2403ZD to AT2404ZD and AT2405ZD, the layout was adjusted, the recognition program was optimized using Stage I data, and the pickling time was extended to 2 min 30 s. After optimization, the first-cycle opening success rate was 15/51 (29.4%), the second-cycle success rate was 16/52 (30.8%), and the cycles 3–8 success rate was 185/201 (92.0%). The detection system identified 216/216 manually confirmed opening events. The improvement should be interpreted as the combined effect of equipment, algorithm, and process optimization, rather than the detection model alone. The event-level control metrics for the two field stages are summarized in Table 10.

### 6.4. Engineering Application Analysis

**Figure 10:**
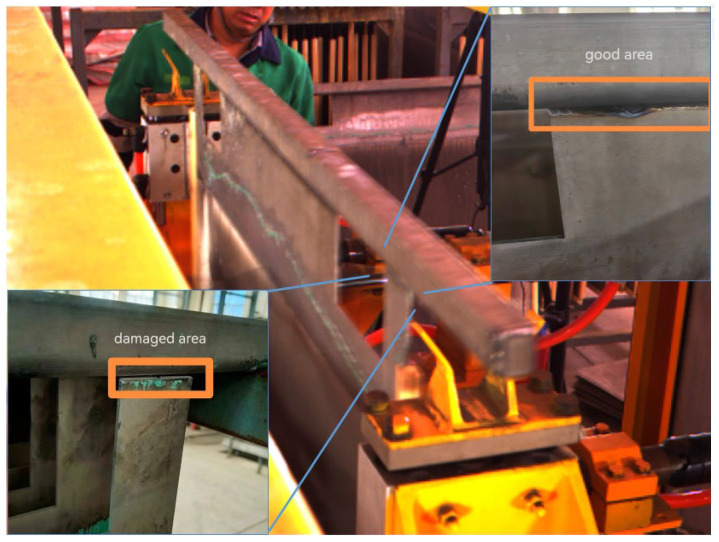
Comparison of visible titanium starter-plate connection damage before and after tamping.

In addition to opening performance, visible damage at the connection between the titanium starter plate and the beam was manually observed during Stage II. A total of 126 plates were inspected, including 104 initially intact plates and 22 plates with pre-existing visible damage. No visually apparent new damage was observed among the 104 initially intact plates. Among the 22 pre-damaged plates, 3 showed visible extension after tamping. A representative comparison of the titanium starter-plate connection before and after tamping is shown in Figure 10. Because this assessment was based on manual visual inspection, it cannot exclude subsurface or sub-millimetre damage. Therefore, under the tested conditions, tamping did not show obvious newly visible damage to initially intact plates, but plates with existing damage still require stricter state assessment and process control.

## 7. Conclusions

This study investigated real-time interface-opening detection for nickel starting sheet pre-stripping under high-frequency tamping. The main conclusions are as follows.

(1) A Yolov11_tapcheck detector was proposed for small, weak-texture, and motion-blurred interface openings. The model retains the lightweight YOLO11n backbone and improves feature representation by using EMA and a BiFPN-inspired bidirectional feature-fusion neck.
(2) A tapcheck dataset was built from field production images. Compared with YOLO11n, the proposed model improved mAP50 from 95.5% to 98.1% and F1 from 93.7% to 97.5%. Ablation results show that EMA and the bidirectional feature-fusion neck both contribute to performance improvement, with the combined configuration achieving the best result.
(3) A real-time detection system was implemented with image acquisition, image detection, production-line control, MQTT communication, OPCSERVER interaction, and PLC control. Logged system verification showed that the frame-to-PLC acknowledgement time remained below the 13.333 ms image acquisition period, meeting the online detection requirement.
(4) Two-stage industrial field tests verified the feasibility of the tamping pre-stripping process and intelligent opening recognition. After coordinated optimization of equipment, recognition software, and pickling time, the cycles 3–8 opening success rate increased from 253/406 (62.3%) to 185/201 (92.0%), and detection success increased from 238/265 (89.8%) to 216/216 (100%).
(5) Under the current test conditions, no visually apparent newly introduced damage was observed among 104 initially intact starter plates, while 3/22 plates with pre-existing damage showed visible extension. Future work will expand the dataset, include more batches and complex operating conditions, and further study long-term equipment stability and starter-plate damage mechanisms.

In addition, the current model is evaluated mainly by detection and control performance, while its internal decision process has not yet been inspected. Future work will use interpretable model-inspection tools, such as Grad-CAM or SHAP, to verify whether the detector focuses on physically meaningful interface-opening boundaries rather than stains, reflections, or irrelevant edges. This direction is consistent with recent work combining real-time prediction and physical interpretability in manufacturing processes [18].

## Figures and Tables

**Table 1:** Relationship of representative methods to the present task.

Method Group	Typical Target	Imaging Condition	Main Strategy	Control Integration	Relevance
YOLO-KD/MGD-YOLO [1,2]	Road defects	Mostly static	Lightweight fusion/attention	No	Small weak targets
YOLO11n-seg/YOLO-ERCD [3,4]	Surface or road cracks	Static images	Segmentation or attention	No	Crack-like textures
OSCD-YOLO/RDLK-YOLO [5,8]	Mine or pipeline defects	Complex background	Partial conv., wavelet, deformable attention	No	Background robustness
FFF thermal monitoring [15]	Manufacturing state	Temporal thermal video	Deep classification	Quality monitoring	Dynamic process state
This work	Interface opening	Motion blur, reflective field scene	YOLO11n + EMA + BFFN	MQTT/OPC/PLC	Event-level closed loop

**Table 2:** Optical and acquisition configuration used in the field system.

Item	Setting	Item	Setting
Camera	Daheng MER2-160-75GC, two units	Frame rate	75 FPS
Raw resolution	1440 × 1080 pixels	Shutter/exposure	Global/20 us
Gain	8 dB	Lens	HN-P-0828-6M-C2/3, 8 mm
Working distance	Approximately 1 m	Lighting	Two 100 W Bull LED floodlights
Trigger mode	Continuous acquisition; software detection window	Field of view	Calculated from lens and working distance
Runtime buffer	Two-slot ping-pong buffer	Deployment	OnnxRuntime.Gpu + MQTT + OPCSERVER

**Table 3:** Dataset composition and leakage-control protocol.

Subset	Opening	Opening-Free	Total	Boxes	Acquisition Groups	Leakage Control
Training	1362	846	2208	1362	114	Event-separated
Validation	292	181	473	292	25	Event-separated
Test	292	182	474	292	24	Event-separated
Total	1946	1209	3155	1946	163	pHash screened

**Table 4:** Reproducible training and inference settings.

Setting	Value	Setting	Value
Input size	640 × 640/letterbox	Batch size	32
Training epochs	200	Optimizer	SGD
Initial LR/final LR factor	0.01/0.01	Warm-up	3 epochs
LR schedule	Default Ultralytics schedule	Augmentation	HSV, translate, scale, flip, mosaic
Momentum/weight decay	0.937/0.0005	Seeds	0, 42, 123, 2025, 3407
Confidence/NMS IoU	0.25/0.70	IoU match	0.50

**Table 5:** Accuracy, complexity, memory, and latency comparison.

Method	P	R	mAP50	mAP50-95	F1	Params (M)	GFLOPs	Size (MB)	GPU (GB)	Latency (ms)
YOLOv8n	0.944	0.932	0.954 ± 0.004	0.682 ± 0.006	0.938	3.2	8.7	6.2	0.74	6.6
YOLOv10n	0.949	0.938	0.959 ± 0.004	0.691 ± 0.006	0.943	2.3	6.7	5.8	0.72	6.5
YOLO11n	0.934	0.941	0.955 ± 0.004	0.698 ± 0.006	0.937	2.6	6.5	5.4	0.71	6.7
YOLO11s	0.965	0.958	0.974 ± 0.003	0.718 ± 0.005	0.961	9.4	21.5	18.4	1.18	9.8
RT-DETR-R18	0.957	0.952	0.968 ± 0.004	0.711 ± 0.006	0.954	20.0	57.0	42.0	1.54	14.9
Yolov11_tapcheck	0.981	0.971	0.981 ± 0.003	0.732 ± 0.005	0.975	3.1	8.2	6.3	0.78	7.1

**Table 6:** Ablation of EMA placement and BFFN.

Configuration	P	R	mAP50	mAP50-95	F1	Params (M)	GFLOPs	Latency (ms)
YOLO11n	0.934	0.941	0.955	0.698	0.937	2.6	6.5	6.7
EMA at P3	0.957	0.951	0.964	0.708	0.954	2.7	6.8	6.8
EMA at P3–P4	0.962	0.955	0.966	0.714	0.958	2.8	7.0	6.9
EMA at P3–P5	0.966	0.957	0.967	0.718	0.961	2.9	7.2	7.0
BFFN only	0.949	0.943	0.965	0.712	0.945	2.8	7.5	6.9
BFFN + EMA at P3–P5	0.977	0.973	0.983	0.730	0.975	3.1	8.2	7.1

**Table 7:** False-positive and false-negative statistics.

Image Type	Samples	Error Type	Count	Rate	Wilson 95% CI
Opening-free	182	False positive	6	3.30%	1.52–7.00%
Opening-containing	292	False negative	8	2.74%	1.39–5.31%

**Table 8:** Model-pipeline and frame-to-PLC acknowledgement latency statistics based on 1000 logged samples.

Timing Item	Mean	SD	Median	p95	p99	Max	Included Components
Model pipeline	7.10	0.34	7.08	7.68	7.91	8.15	preprocessing, GPU inference, post-processing
Frame-to-PLC acknowledgement	10.24	0.46	10.21	11.03	11.34	11.58	model pipeline, MQTT transmission, OPCSERVER writing, PLC feedback timestamp, 1 ms polling

**Table 9:** Exact counts and opening outcomes in the two field stages.

Stage	Period	Sheets	Cycle 1 Group	Cycle 2 Group	Cycles 3–8 Group	Detected/Confirmed Openings	Main Conditions
I	27 August–26 September 2025	612	3/102 (2.9%)	9/104 (8.7%)	253/406 (62.3%)	238/265 (89.8%)	Original equipment/process/software
II	27 October–17 November 2025	304	15/51 (29.4%)	16/52 (30.8%)	185/201 (92.0%)	216/216 (100%)	Larger tampers, adjusted layout, updated software, 150 s pickling

**Table 10:** Event-level control metrics for the two field stages.

Metric	Stage I	Stage II	Definition
First-detection delay	23.8 ± 8.4 ms	18.6 ± 6.1 ms	First ground-truth opening frame to first accepted prediction
Missed opening events	27/265 (10.2%)	0/216 (0%)	No accepted prediction within 50 ms
Premature-stop events	4/612 (0.65%)	0/304 (0%)	Stop before observer-confirmed opening
Successful stop commands	234/238 (98.3%)	216/216 (100%)	PLC acknowledgement after valid detection

## Data Availability

The data that support the findings of this study are available upon request from the corresponding author.
